# Tuberculosis and HIV co-infection in children

**DOI:** 10.1186/1471-2334-14-S1-S5

**Published:** 2014-01-08

**Authors:** Elisabetta Venturini, Anna Turkova, Elena Chiappini, Luisa Galli, Maurizio de Martino, Claire Thorne

**Affiliations:** 1 Department of Health Sciences, Meyer Children University Hospital, University of Florence, Florence, Italy; 2 Department of Paediatric Infectious Diseases, St Mary's Hospital, Imperial College NHS Trust, London, United Kingdom; 3 Centre of Paediatric Epidemiology and Biostatistics, University College London Institute of Child Health, London, WC1N 1EH, United Kingdom

## Abstract

HIV is the top and tuberculosis is the second leading cause of death from infectious disease worldwide**,** with an estimated 8.7 million incident cases of tuberculosis and 2.5 million new HIV infections annually. The World Health Organization estimates that HIV prevalence among children with tuberculosis, in countries with moderate to high prevalence, ranges from 10 to 60%. The mechanisms promoting susceptibility of people with HIV to tuberculosis disease are incompletely understood, being likely caused by multifactorial processes.

Paediatric tuberculosis and HIV have overlapping clinical manifestations, which could lead to missed or late diagnosis. Although every effort should be made to obtain a microbiologically-confirmed diagnosis in children with tuberculosis, in reality this may only be achieved in a minority, reflecting their paucibacillary nature and the difficulties in obtain samples. Rapid polymerase chain reaction tests, such as Xpert MTB/RIF assay, are increasingly used in children. The use of less or non invasive methods of sample collection, such as naso-pharyngeal aspirates and stool samples for a polymerase chain reaction-based diagnostic test tests and mycobacterial cultures is promising technique in HIV negative and HIV positive children. Anti-tuberculosis treatment should be started immediately at diagnosis with a four drug regimen, irrespective of the disease severity. Moreover, tuberculosis disease in an HIV infected child is considered to be a clinical indication for initiation of antiretroviral treatment. The World Health Organization recommends starting antiretroviral treatment in children as soon as anti-tuberculosis treatment is tolerated and within 2- 8 weeks after initiating it. The treatment of choice depends on the child’s age and availability of age-appropriate formulations, and potential drug interactions and resistance. Treatment of multi-drug resistant tuberculosis in HIV-infected children follows same principles as for HIV uninfected children. There are conflicting results on effectiveness of isoniazid preventive therapy in reducing incidence of tuberculosis disease in children with HIV.

**Conclusion:**

Data on HIV/TB co-infection in children are still lacking. There are on-going large clinical trials on the prevention and treatment of TB/HIV infection in children that hopefully will help to guide an evidence-based clinical practice in both resource-rich and resource-limited settings.

## Global epidemiology of HIV and TB

Worldwide, 2 billion people are estimated to be latently infected with tuberculosis (TB) [1], whilst there are an estimated 35.3 million people living with HIV, 70% of whom live in sub-Saharan Africa (SSA) [2]. HIV is the top and TB is the second leading cause of death from infectious disease worldwide [1]. Annually there are an estimated 8.7 million (range, 8.3-9.0 million) incident cases of TB and 2.5 million new HIV infections [1,2]. Of the 8.7 million incident cases of TB in 2011, 1.1 million (13%) were among people living with HIV [2]. There are well established epidemiological and biological synergies between HIV and TB, influencing the distribution, progression and outcomes of both infections. The HIV epidemic is a key factor behind the resurgence in TB incidence worldwide and HIV is the pre-eminent risk factor for the development of TB. One in eight incident cases of TB occur in HIV positive individuals, with a quarter of all TB deaths in people with HIV, while around a fifth of HIV related deaths occur in incident TB cases [2,3]. Much of the burden of TB and HIV co-disease is in Africa, where one-third of the approximately 2.3 million people who developed TB in 2010 were HIV positive [1], although other regions strongly affected by TB and HIV dual epidemics are India and Eastern Europe [4,5].

The HIV epidemic has also had an impact on the age related prevalence of TB disease, with a lowering in the peak age groups affected, such that in high HIV prevalence areas, the peak TB incidence rates are now seen among adults aged 20-45 years [6].

## Children and HIV

The impact of the HIV pandemic on children has been huge, and children account for around 10% of new global HIV infections with an estimated 3.4 million children aged less than 15 years living with HIV in 2010 [7]. The survival of children in antiretroviral treatment (ART) era has dramatically improved in both resource-rich and resource-limited settings [8-12]. Although coverage with ART is improving, it is estimated that in low and middle income countries only around 34% of children aged <15 years needing ART are estimated to be receiving treatment, compared with 68% in adults [2,7,13].

## Epidemiology of paediatric HIV/TB disease

There are scarce data on the incidence of TB among HIV positive children and the prevalence of HIV among children diagnosed with TB, and the information that is available is difficult to interpret due to problems with diagnosis, under-ascertainment and selection of study populations (i.e. most are recruited from hospitals or referral hospitals rather than the community). The World Health Organization (WHO) estimates that HIV prevalence among children with TB, in countries with moderate to high prevalence, ranges from 10 to 60% [7], with the prevalence varying depending on the background rates of HIV infection and estimates from several studies are summarised in Table 1[14-21].

**Table 1:** Prevalence of HIV among children with TB [14-21]

Study setting	Year	Study population	HIV prevalence	Reference
Zambia	1994	Children with clinical TB diagnosis (n=120)	56%	Luo et al *Tuberc Lung Dis* 1994 [14]

Johannesburg, South Africa	2008	Cultured confirmed cases of TB in children aged <14 years (n=1317)	52% in children with drug-susceptible TB; 53% in the 13 with MDR-TB	Fairlie et al *BMC Inf Dis* 2011[15]

Mumbai, India	2002	Children with disseminated TB (n=68)	16%	Karande et al *J Trop Pediatr* 2002 [16]

Rio de Janeiro, Brazil	1999-2008	Children attending a reference hospital (n=473)	17% (but only 56% tested for HIV)	Matos et al *J Pediatr* 2012 [17]

Cape Town, South Africa	1999-2004	Children born in 1999 and diagnosed with TB in public health facilities up to 2004 (n=1607)	37% (but only 16% had HIV test results available)	Moyo et al *Int J Tuberc Lung Dis* 2010 [18]

Santo Domingo, Dominican Republic	1996	Children aged 18-59 months with clinical TB diagnosis (n=189)	5.8%	Espinal et al *J Acquir Immune Defic Syndr Hum Retrovirol* 1996 [19]

Durban, South Africa	1998-1999	Hospitalised children with culture-confirmed TB aged <13 years (n=118)	48%	Jeena et al *Int J Tuberc Lung Dis* 2002 [20]

Abidjan, Côte d’Ivoire	1994-1995	Children aged 0-9 years with newly diagnosed TB (out- and in-patients) (n=161)	19%	Mukadi et al *AIDS* 1997 [21]

Estimated rates of TB among children with HIV also vary widely, partly depending on whether the study is taking place in a TB endemic area or not and on highly active anti-retroviral treatment (HAART) coverage in that area, but also due to the problems of reaching a definitive diagnosis of TB in children with HIV and under-ascertainment. In one large paediatric HIV clinic in London, UK, there was an average of two children with HIV per year who presented with active TB over a 15 year period (18/328, 5.5% of HIV infected patients were treated for active TB) [22] and in a US cohort of nearly 1500 HIV infected children, TB disease was found 3%, with an incidence of 0.61 per 100 child-years [23]. In contrast, in high prevalence countries, the incidence of TB disease in HIV positive children is much higher. Thus, in a South African retrospective study, incidence of TB disease was estimated to be 23 per 100 child-years among HIV positive children receiving HIV care [24]. With increasing coverage with ART, the incidence of TB has been decreasing, but remains substantially higher in HIV positive children than in the general paediatric population [25].

## Impact of HIV on pathogenesis of TB

HIV alters the pathogenesis of TB, increasing the risks of developing active TB in those with latent infection as well as in those newly exposed to TB. In the HIV-uninfected population, only around 10% of people infected with TB will develop TB disease [1]. However, in HIV positive people, there is a 20-30 fold increased relative risk of developing TB disease from latent state compared with that in people without HIV [3], an increase that outweighs those of other risk factors such as malnutrition. The mechanisms promoting susceptibility of people with HIV to TB disease are incompletely understood. Risk of active TB increases with depletion of CD4 T-cells, but studies have also shown that HIV positive individuals in high TB incidence regions have an increased risk of developing active TB in the first year after HIV seroconversion, i.e. with high CD4 T-cell counts [26]. In addition, HIV positive individuals on ART with high CD4 T-cell counts continue to have an increased risk of developing TB compared with uninfected controls. This suggests that although the loss of cell-mediated immunity with HIV disease progression is likely to be an important factor with respect to the increased risk of active TB associated with HIV, this is likely to be caused by multifactorial processes.

Studies have demonstrated depletion of TB-specific CD4 T cells in peripheral blood and in the lung at early stages of HIV disease, suggesting that TB-specific adaptive immunity may be especially susceptible to HIV-associated immune damage [27]. The essential role of CD4 T cells in generation of granulomas, and the depletion of such cells with HIV disease progression, may explain the increased risk of extra-pulmonary TB (EPTB) in HIV-positive patients [28]. Furthermore, the failure of the declining CD4 T cell population to regulate and maintain granulomas is one of the mechanisms proposed to be behind the increasing risk of re-activation disease in latently infected individuals with HIV. Turning to innate immunity, apoptosis (programmed cell death) of an infected macrophage is an important host immune response to TB infection. Recent work has suggested that in the context of HIV infection, apoptosis of alveolar macrophages is decreased, possibly due to raised IL-10 (an anti-inflammatory cytokine) in the lung [29], which may be a mechanism behind the increased susceptibility of those with HIV to TB. Genetic variants are also known to influence TB development in HIV positive patients. A recent case-control study in Brazil showed the novel association between certain inflammasome gene polymorphisms (i.e. CARD8 genetic variants) and the development of TB infection in HIV positive subjects [30].

## Challenges of diagnosis of TB in children with HIV

WHO guidelines state that diagnosis of TB in an HIV positive child should follow the same approach as for HIV-uninfected children, with taking into account the history of TB contact, clinical features suggestive of TB (such as prolonged cough, poor weight gain or weight loss), positive tuberculin skin test (TST) ≥5mm considered to be positive in HIV infected individuals, and suggestive chest X-ray signs [31].

Paediatric TB and HIV have overlapping clinical manifestations, including fever, weight loss and lymphadenopathy, which combined with persistent cough, could lead to missed or late diagnosis, or alternatively a misdiagnosis of either infection or concomitant infections with similar clinical features. TB generally has a more severe clinical presentation in HIV positive individuals than in those uninfected. The risk of EPTB or disseminated TB is increased in HIV positive adults, particularly with low CD4 T cell counts [28]. In a US study of HIV positive adults with EPTB, there was a significantly increased risk of central nervous system, meningeal or disseminated TB disease compared to lymph node disease in those with severe immunosuppression (CD4 <100 cells/mm^3^) [32]. In the limited paediatric literature, studies have reported no significant differences in the frequency of EPTB between HIV positive and negative children [20,21,33]. However, others have suggested that HIV infected children with more advanced HIV disease are at higher risk of EPTB and combination of extrapulmonary and pulmonary TB disease [34,35]. Overall, the clinical presentation of TB in HIV infected children, may depend on degree of immunocompromise, with severe, disseminated forms more frequently found in patients with advanced HIV infection; however this link is less well reported than in adults.

The presence of HIV co-infection compounds the well-recognised challenges of reaching a definitive diagnosis in children with suspected TB. Although every effort should be made to obtain a microbiologically-confirmed TB diagnosis in children, in reality this may only be achieved in a minority, reflecting the paucibacillary nature of much childhood TB, the inability of young children to expectorate sputum, the difficulties of obtaining gastric aspirates and the low yield of such samples. Sputum induction with hypertonic saline was found to be safe and useful for microbiological confirmation of pulmonary TB in both HIV positive and HIV negative children in a study in South Africa: overall, 24% of children had a positive smear or culture for *Mycobacterium tuberculosis* and induced sputum (IS) samples had a three-times greater yield than gastric lavage (GA), with no difference in yield by HIV infection status [36]. This was later evaluated in the community setting with 16.9% of clinically diagnosed TB cases confirmed microbiologically [37], highlighting the usefulness of this diagnostic tool in the outpatient settings. However the need for electrical supply and special technical equipment, together with the high work load and infectious hazard are substantial obstacles for widespread use of this method. It is important to note that low rates of microbiological confirmation in paediatric TB are in part due to low rates of obtaining the samples from children. For example, European Centre for Disease Prevention and Control (ECDC) surveillance demonstrated that only 42% of children with TB reported to ECDC had mycobacterial cultures sent in 2000-2009, however of those who had their cultures sent, 40% were culture positive [38]. It is important to highlight that every effort needs to be undertaken to collect samples from children with suspected TB for microbiological confirmation.

Rapid polymerase chain reaction (PCR) tests, such as Xpert MTB/RIF assay, are increasingly used in children with TB and are recommended by WHO as the initial diagnostic test in patients suspected of MDR-TB or HIV/TB [39]. The test is an important advance in rapid detection of TB disease and detection of drug resistance. The few prospective studies evaluating it in children have showed that it is much more sensitive than microscopy, with sensitivity being reported from 75 to 90% on sputum samples and nearly 70% on gastric aspirates, with comparable performance in HIV positive and HIV negative children [40,41]. Although the sensitivity of Xpert MTB/RIF test is higher than microscopy, a substantial proportion of children with negative test had positive MTB cultures. Hence Xpert MTB/RIF test cannot be used to rule out TB, and MTB culture remains a necessary diagnostic tool.

The use of less or non invasive methods of sample collection, such as naso-pharyngeal aspirates (NPA) and stool samples for a PCR-based diagnostic test tests and MTB cultures is promising technique which was evaluated in HIV negative and HIV positive children. Small pilot studies in South Africa reported on Xpert MTB/RIF assay on stool in childhood TB. The first study assessed Xpert on decontaminated stool sediment. It showed that stool Xpert was equally sensitive to the assay on GA, detecting 75% of children with intra-thoracic culture confirmed TB [42]. In another pilot study, stool Xpert, which was performed directly on stool detected 47% culture-confirmed TB (80% in HIV-infected children and 33% in HIV-uninfected children) compared to 65% cases detected by Xpert on IS [43]. The study used small stool volumes, and the authors concluded that the sensitivity may be increased by an improved protocol. Another paediatric study from the South Africa compared NPA with IS: the sensitivity of two Xpert MTB/RIF tests were comparable (71% vs 65%, P = 0.444), however the culture yield from NPA was significantly less than from IS (96.6% vs 70.1%, P < 0.001) [44]. Stool and NPA can be useful add on or alternative specimens in the settings where is unavailable and in older children in whom GA are difficult to obtain. Urine lipoarabinomannan has been recently shown to be useful in diagnosing TB in severely immunocompromised adults, detecting up to 61% of culture-confirmed TB cases with CD4<50 cells/mm^3 ^[45]. There are no reported studies in children to date.

With respect to TST, there is a high rate of false negative results in HIV positive children. In a study in Cape Town, among nearly 300 paediatric TB cases, those with HIV were significantly less likely to have a positive TST than those HIV negative (36% versus 59%) [18]. In a further study in South Africa among HIV-infected children with culture-confirmed TB, only 56% had a positive TST [35]. There is considerable discrepancy between current recommendations on use of interferon-γ release assays (IGRA) and TST in diagnosis of TB infection and TB disease in children [46]. A systemic review and meta-analysis on IGRA in childhood tuberculosis showed improved specificity of IGRA compared with TST and similar accuracy between IGRA and TST in detection of TB infection or TB disease; however the tests showed high level of discordant results and had lower sensitivity for HIV positive children [47]. In a recent South African study comparing performance of the TST and IGRA in children recruited from hospital outpatient settings, while the proportion with a positive TST did not vary according to HIV infection status, HIV positive children were significantly less likely to have a positive IGRA than HIV negative children after adjusting for degree of TB exposure; in addition, HIV negative children had an increasing probability of a positive TST with increasing age compared to HIV positive children, after adjusting for degree of TB exposure, Bacillus Calmette–Guérin (BCG) vaccination, malnutrition and past TB [48]. The authors concluded that caution is needed in interpreting IGRA results in the context of HIV infection, given their findings that the test performance compared with TST is differentially affected by age and HIV infection. Some studies demonstrated that enzyme-linked immunospot assay is more sensitive than TST in detection of active TB in HIV positive children however the sensitivity was not sufficiently high to rule out active TB [49,50].

Although in adults with TB and HIV, particularly those with severe immunodeficiency, there may be atypical chest X-ray findings, the limited studies in children have not identified key differences in the radiological presentation of TB according to HIV status [20,51,52]. HIV positive children may frequently have other respiratory opportunistic infections such as *Pneumocystis jirovecii* pneumonia, lymphoid interstitial pneumonitis or other bacterial or viral pneumonias, which can further complicate diagnosis of pulmonary TB, for example, due to similar clinical or radiological manifestations. However, the presence of one such opportunistic infection does not preclude concurrent infection with another, and the challenge is to identify all respiratory infections present and to treat appropriately.

In a case series of 18 children with HIV and active TB in London, nearly half of the children had not yet been diagnosed with HIV at the time they presented with TB [22]. The new directions in diagnosis of paediatric TB and novel TB diagnostic tests which are under development have been recently reviewed by Whittaker and colleagues [53].

## Clinical management of HIV/TB in children

### Prognosis

Many paediatric studies reported that children experiencing HIV/TB co-disease have more severe disease and higher mortality than HIV negative children, reflecting the dual impact of the two infections. In the pre-ART era, very high mortality rates were reported for children with TB and HIV: in a retrospective study of HIV-infected children with culture confirmed TB in South Africa, 21% of children died whilst on TB therapy and a further 18% after TB treatment was competed, mostly (in 82% cases) due to causes other than TB [35]. In a cohort study in Ethiopia, children with HIV infection were around six times more likely to die from TB than those without HIV [52]. In a study in Côte d’Ivoire, the mortality rate in HIV-infected children with TB was 23% versus 4% in HIV-uninfected children, a 3.6 times increased risk; of note all deaths among the children with HIV occurred in those with severe immune suppression [21]. Not all paediatric studies showed higher TB associated mortality in HIV co-infected children: in a Zambian autopsy study of 180 HIV positive and 84 HIV negative children who died of respiratory disease, 26% of the HIV-negative and 18% of the HIV positive group died with TB (no significant difference), half of the HIV positive children had more than one respiratory disease or infection identified [54].

ART has modified HIV/TB co-infection epidemic and had a great impact on TB disease incidence, morbidity and mortality. In a study in the Democratic Republic of Congo, ART halved the hazard of developing TB in HIV-infected children [55], with a TB incidence rate in those receiving ART of 10.2 per 100 person-years versus 20.4 per 100 person-years in those not yet on ART; after 12 months of ART TB incidence decreased to 5.3 per 100 person-years. A recent prospective cohort study conducted in Kenya on 689 HIV-infected children aged 6 weeks to 14 years, showed that longer time on ART and longer duration of being in care before ART were associated with lower prevalence of TB at enrolment (adjusted hazard ratio [aHR] 0.91, P = 0.003 and aHR 0.87, P= 0.001 respectively) and lower incidence of TB during the study (aOR 0.91, P< 0.001 and aOR 0.92, P < 0.001 respectively) [56]. These findings are consistent with those from a South African retrospective cohort study that reported that among children receiving ART, incidence of clinically diagnosed TB declined from 21.1 per 100 person-years during their pre-ART follow-up to 6.4 per 100 person-years after ART initiation [57]. A recent retrospective study from Soweto, South Africa, showed that scaling-up of the public-funded antiretroviral treatment program resulted in 70.6% reduction of culture-confirmed TB in HIV infected children in 4-year period from 2005 to 2009. However in 2009 the incidence of culture confirmed TB, was still 42-fold times higher in HIV positive compared to HIV negative children (460.7/100,000 vs. 11.0/100,000). Over three quarters of co-infected children were severely immunocompromised [58]. Over 40% decline of TB incidence was also observed in HIV negative children which maybe in part due to increased ART coverage in adults and the associated decreased TB transmission.

### Treatment

Anti-TB treatment should be started immediately at TB diagnosis. In the most recent guidance, WHO recommends that all HIV positive children are started on four-drug TB treatment, irrespective of the TB disease severity (isoniazid, rifampicin, pyrazinamide and ethambutol) and that intermittent therapy should be avoided [13,59].

TB disease in a HIV infected child is considered to be a clinical indication for initiation of ART [59]. WHO 2013 recommends starting ART in children as soon as TB treatment is tolerated and within 2- 8 weeks after initiating it [59]. The early start of ART is especially relevant for children with moderate-to-severe immunocompromise. The recommendations are extrapolated from adult trials, which showed that mortality is decreased in severely immunocompromised patients with CD4 <50 cells/mm^3^ with early start of ART [60-62]. A paediatric retrospective study from Johannesburg, South Africa, in children, most of whom were severely immunocompromised, showed that delaying ART for longer than 8 weeks was associated with increased mortality and worse virologic outcome [63]. The data from adult studies suggest that delayed ART in patients with no or mild immunocompromise was not associated with worse outcomes [60-62]. Therefore some clinicians consider delaying ART in children with no evidence of immunocompromise until TB treatment is well tolerated or even until the end of TB treatment in order to avoid potential drug interactions and significantly increased pill burden [22].

The choice of ART depends on the child’s age and availability of age appropriate formulations, whether the child is already receiving treatment, their previous ART exposure, and potential drug interactions. Significant drug interactions occur with rifampicin, which is a potent CYP3A4 inducer, with a co-administration with nevirapine and protease inhibitors which are metabolised through cytochrome C450 enzymes. This may lead to a marked decrease in plasma concentration of these drugs; therefore in order to maintain therapeutic levels dose adjustment may need to be done when starting and stopping rifampicin. The regimen of choice in children aged over 3 years is efavirenz (EFV)-based ART, as EFV has less significant drug interaction with rifampicin and dose adjustment is not required. EFV capsule sprinkles have been recently licensed in children aged over 3 months based on results of a paediatric population model [64]. However EFV capsules are not widely available in high HIV and TB prevalence settings, and further studies are required to assure the efficacy of EFV-based combinations in HIV/TB co-infected young children.

If lopinavir/ritonavir based ART is used, to overcome drug interactions with rifampicin, it will be necessary to increase ritonavir to 1:1 of lopinavir/ritonavir [65,66]. In settings where therapeutic drug level monitoring is available, dose adjustment of rifampicin and lopinavir or other protease inhibitors can be made based on the results. If available, rifabutin, a less potent CYP3A4 inducer, should be used instead of rifampicin to reduce drug interactions. ART-naïve children under 3 years of age can be started on nevirapine-based ART but without lead-in dose and maximum recommended dose (200mg/mm^3^ twice daily) [59]. Some clinicians choose to increase the maintenance dose of nevirapine by 20-30% in order to reach an optimal exposure [67].

An alternative regimen in children is triple nucleoside reverse transcriptase inhibitors administered for the duration of anti-TB treatment [59]. This regimen has less virological efficacy but no significant drug interaction with rifampicin. It has been shown in a recent paediatric trial in Uganda and Zimbabwe to be immunologically and clinically similar to non-nucleoside reverse transcriptase inhibitors based ART and therefore may be valuable in children with controlled HIV infection who develop TB [68]. Information on drug interactions and advice on dose modification can be found at http://www.hiv-druginteractions.org.

There remains some debate with respect to duration of TB therapy in HIV-infected children. There is no evidence that HIV-infected children should have longer duration of treatment than HIV uninfected children if a good response to treatment is achieved. Generally HIV infected children without severe TB disease can receive standard duration of anti-TB treatment for 6 months, and the duration of anti-TB treatment can be extended in cases with suboptimal response to treatment [31]. However in the latter cases, poor adherence, suboptimal drug levels secondary to insufficient dosing or drug interactions, drug resistance and immune reconstitution inflammatory response should be considered and addressed appropriately.

### Multi drug resistant (MDR)-TB

There are limited data on the extent of MDR-TB among HIV positive children. In one study in the Western Cape Province, South Africa, 5% of HIV-infected children aged <14 years with culture confirmed TB had MDR-TB [35]. A cross sectional study in children with culture confirmed TB in a setting with high prevalence of HIV (Johannesburg, South Africa) showed no difference in HIV prevalence in TB compared to MDR-TB (52.1% vs. 53.9% respectively), suggesting there is no association between HIV infection and MDR-TB [15]. MDR-TB can be devastating in HIV infected individuals as demonstrated by the very high mortality rates in HIV positive adults in early institution-based outbreaks with MDR-TB and extensively drug resistant (XDR)-TB [69]. Advances in treatment of both HIV and drug resistant TB, combined with comprehensive care, have facilitated very good treatment outcomes in HIV infected children with MDR-TB and even XDR TB [70,71]. A retrospective study from Western Cape Province, South Africa, on children with culture-confirmed MDR-TB, with HIV prevalence of 43%, overall treatment success was achieved in 82%; multivariable analysis showed no significant association of HIV infection with poor treatment outcome (OR 1.46 [95% CI, 0.46–4.63]; P=0.52) [72].

Treatment of MDR-TB in HIV infected children follows the same principles as for HIV uninfected children as well outlined in the recent guidelines [73-75]. Based on extrapolations from adult studies, children with MDR-TB are recommended to receive TB treatment for 18-24 months regardless of their HIV status [75]. However, good results have been demonstrated with shorter and less intensive regimens in children with non-severe forms of TB disease [76]. Children with HIV/TB co-infection should be monitored closely for potential overlapping and added toxicities of ART and second line TB drugs. Attention to nutrition and adherence support with provision of directly observed therapy are extremely important.

### Immune reconstitution inflammatory syndrome (IRIS)

Following initiation of ART in HIV/TB co-infected patients, IRIS may develop - presenting with either clinical manifestation of previously undiagnosed TB or paradoxical exacerbation of already diagnosed TB disease in spite of already initiated anti-TB treatment [77]. It usually occurs in severely immunocompromised patients and presents within 3 months of starting ART. It is frequently associated with a substantial fall of viral load and increase in CD4 count. Non-inflammatory drugs in mild cases or steroids in moderate-to-severe cases are usually required in the addition to perseverance with TB and HIV treatment. Most patients can be managed without stopping ART.

## Perinatal TB/HIV

African studies carried out in the pre-ART era demonstrated that recent pregnancy was associated with increased risk of developing active TB in HIV positive women, which is associated with increased risk of maternal mortality [78]. Infants of mothers with active TB are at high risk of acquiring TB infection (usually postnatally) and developing active disease [79]. The extrapulmonary forms of the disease, miliary and meningeal TB, are greater risk factors for congenital TB [80]. In one study, at least 15% of mothers with active TB in pregnancy had transmitted infection to their infants by age three weeks [81]. In an Indian study of HIV-infected postnatal women not receiving isoniazid TB preventative therapy (IPT), there was a high maternal active TB incidence (5 cases/100 person-years), and infants of mothers with incident TB had a 3-fold increased mortality rate compared with other infants [82]. Transmission and outcomes are affected by duration of therapy before delivery. Four months or more of therapy is protective to the foetus. However, noncompliance with therapy carries an increased risk of transmission of the *Mycobacterium tuberculosis* to the infant [83]. Further research to guide policies for TB screening and IPT in pregnant women with HIV is needed.

## Prevention

### Isoniazid preventive therapy

There are controversial results on effectiveness of TB preventive therapy in reducing incidence of TB disease in children with HIV. Zar and colleagues in a South African clinical trial including nearly 300 children showed that IPT halved mortality (8% versus 16%) and reduced TB incidence from 10% to 4%, reducing the chance of developing TB (confirmed or probable) by 72% [84]. However a recently conducted large double-blind, randomized, placebo-controlled trial of pre-exposure isoniazid prophylaxis against tuberculosis on 548 HIV infected children and 804 HIV uninfected children immunized with BCG vaccine, showed no significant difference in the combined incidence of tuberculosis infection, tuberculosis disease, or death between the isoniazid group and the placebo group irrespective of HIV status [85].

The differences in coverage of ART and lack of exposure to TB disease are the likely explanations for the contrasting results, which was further underlined by Mark Cotton of Stellenbosch University who participated as an investigator in both trials [84-86].

Recently, results on the use of 3 months of rifapentine and isoniazid once weekly for people living with HIV showing higher treatment completion rates and better tolerance than 9 months of isoniazid administered daily. This regimen was as well tolerated in HIV-infected as in HIV negative individuals. Although increased risk of selection for rifampicin resistance was observed in the rifapentine/isoniazid group, the numbers were too few for conclusions to be drawn [87].

### BCG and the HIV-infected Infant

While the risks of adverse events associated with BCG vaccination in HIV negative infants are low (<0.04% for local disease and 0.0002% for disseminated disease), HIV positive infants have a markedly increased risk of developing local (5.6%) and disseminated BCG disease (dBCG) (0.2%) [87]. Recently the WHO assessed the risk of dBCG disease in HIV-positive infants to be approaching 1% [31]. Moreover HIV infection severely impairs the BCG-specific T cell responses during the first year of life, giving little protection against tuberculosis in HIV-infected infants. Considering the significant risk of dBCG disease, these data strongly support the WHO recommendation of not giving BCG to children who are known to be infected with HIV [31]. However, in settings where limited resources are available, BCG vaccination is given at birth to all infants regardless of HIV exposure, considering the high endemicity of tuberculosis in populations with high HIV prevalence. Close follow up of infants known to be born to HIV-infected mothers and who received BCG at birth is recommended in order to provide early identification and treatment of any BCG-related complication [31].

## Conclusion

TB and HIV both represent major threats to public health worldwide. Renewed global interest led to advances in recognising TB/HIV co-infection and understanding the mechanisms promoting susceptibility of HIV-infected people in developing TB disease as part of a multifactorial process [26-30]. Diagnosis of HIV/TB co-infection in children is still challenging. Paediatric TB and HIV have overlapping clinical manifestations, which could lead to missed or late diagnosis. TB disease in a child should alert the clinician to the possibility of HIV infection, particularly in high HIV prevalence settings, where HIV counselling and testing to the child and family should be strongly recommended. Difficulties in TB diagnosis in children are mostly related to their paucibacillary nature. New tests based on PCR have been developed, being applied in samples that require less invasive procedures such as stool and NPA and showing to be useful especially in settings in whom GA are difficult to obtain. Larger studies are needed to establish the sensitivity of these tests to rule out TB in children [42-44]. Few data are available about IGRA and TST in HIV positive children, showing high level of discordant results and lower sensitivity in these patients. Further well-designed and comparative studies on use of IGRA in immunocompromised children are required [46-50].

Following WHO 2013 recommendations, anti-TB treatment should be started immediately after diagnosis whereas ART could be delayed to 2-8 weeks after or as soon as TB therapy is tolerated. The regimen of choice should be carefully addressed considering age, drug interactions, especially if rifampicin is used, or MDR-TB cases [13,59].

There are controversial results on effectiveness of preventive therapy in reducing incidence of TB disease in HIV children. The last CROI meeting in 2013 reported that primary isoniazid prophylaxis seemed ineffective in preventing TB infection in young HIV-perinatally exposed infants (<12 months) in TB endemic regions [86]; nevertheless treatment of latent TB with isoniazid was effective in avoid developing TB disease in HIV infected children over 24 months of age [86]. Thus the questions remain regarding the protective effect of IPT in low TB burden settings. Moreover, shorter regimens have been studied in adults, i.e. 3 months rifapentine and isoniazid once weekly, with promising preliminary results on efficacy and compliance [87]. There are on-going large clinical trials (i.e. the PREVENT TB study, SHINE-trial) on the prevention and treatment of TB/HIV infection in children that should provide new, much needed data on paediatric TB/HIV infection and help to guide evidence-based clinical practice in both resource-rich and resource-limited settings.

## List of abbreviation used

ART: anti-retroviral treatment; BCG: bacillus Calmette–Guérin; dBCG: disseminated BCG disease; ECDC: European Centre for Disease Prevention and Control; EFV: efavirenz; EPTB: extra-pulmonary TB; GA: gastric aspirate; IGRA: interferon-γ release assays; IPT: Isoniazide preventing therapy; IRIS: Immune reconstitution inflammatory syndrome; IS: induced sputum; MDR: multi drug resistant; NPA: naso-pharyngeal aspirate; PCR: polymerase chain reaction; SSA: sub-Saharan Africa; TB: tuberculosis; TST: tuberculin skin test; WHO: World Health Organization; XDR: extensively drug resistant

## Competing interests

The authors declare that they have no competing interests

## Declarations

Funding for this supplement came from the Italian Health Ministry /Young Research Project.

This article has been published as part of *BMC Infectious Diseases* Volume 14 Supplement 1, 2014: Highlights in Pediatric Tuberculosis. The full contents of the supplement are available online at http://www.biomedcentral.com/bmcinfectdis/supplements/14/S1
